# Is Obesity a Risk Factor for Periodontitis? A Systematic Review and Meta‐Analysis

**DOI:** 10.1111/obr.70020

**Published:** 2025-09-16

**Authors:** Fariba Esperouz, Domenico Ciavarella, Claudio Di Gioia, Gaetano Serviddio, Mauro Lorusso, Lucio Lo Russo

**Affiliations:** ^1^ Department of Clinical and Experimental Medicine, School of Dentistry University of Foggia Foggia Italy; ^2^ Private practice Bari Italy; ^3^ Department of Medical and Surgical Sciences University of Foggia Foggia Italy

**Keywords:** Body Mass Index, BMI, obesity, oral health, periodontitis, prevention, probing depth

## Abstract

**Objectives:**

This study aims at reviewing available evidence on the relationship between obesity and periodontitis, focusing on whether obesity is a risk factor for the development and progression of periodontal disease.

**Materials and Methods:**

This systematic review and meta‐analysis were reported in accordance with the PRISMA guidelines. Searches were performed in databases such as PUBMED, SCOPUS, and Web of Science for studies investigating the correlation between obesity (BMI ≥ 30 kg/m^2^) and periodontitis in adult patients. Heterogeneity was assessed using the *I* ^2^ statistic, and a meta‐analysis was performed to calculate pooled odds ratios with 95% confidence intervals.

**Results:**

The analysis included 19 studies with a total of 41,107 patients. The meta‐analysis showed a significant association between obesity and an increased risk of periodontitis (OR = 1.31 95% CI: 1.22–1.41) confirming that obesity is a risk factor for periodontal disease.

**Conclusion:**

The results suggest that obesity is significantly associated with an increased risk of periodontitis. Given this correlation, further research is needed to better understand the implications of this association on clinical outcomes.

## 1 Introduction

In the past 50 years, obesity prevalence increased worldwide: the percentage of adults with obesity 18 years of age or older more than doubled, rising from 7% to 16% [1, 2]. Obesity, assessed using the Body Mass Index (BMI > 30 kg/m^2^), may impact physical and mental health, leading to a poorer quality of life [3] and causing or aggravating chronic conditions such as type II diabetes, dyslipidemia, hypertension [4], cardiovascular diseases, cancers, neurological disorders, chronic respiratory diseases, and digestive disorders [5].

Periodontitis is a chronic inflammatory disease with a complex, multifactorial etiology, primarily associated with dysbiotic microbial biofilms that form around the teeth [6]. This condition leads to the progressive destruction of teeth supporting tissues, including the gums, periodontal ligament, and bone [6], thus causing detrimental effects on oral functions, as well as on quality of life.

From a public health perspective, the burden of periodontal diseases is significant: Periodontitis is one of the most widespread chronic diseases globally [7]. Many studies [8, 9, 10] and the Third National Health and Nutrition Examination Survey (NHANES III) [11] have confirmed its high prevalence and its substantial impact on oral and general health [12]. The rising prevalence of both obesity and periodontitis represents a substantial challenge for healthcare systems worldwide [13]. Recent data show that, between 2011 and 2020, periodontitis affected approximately 62% of dentate adults, with 23.6% presenting with severe disease [14]. Their impact on health outcomes, the socioeconomic burden of obesity [15], and periodontal disease [16] is considerable. Both conditions are associated with elevated healthcare costs, and their combined prevalence can result in significant productivity losses due to disability and reduced quality of life [17, 18]. In patients with obesity, elevated blood levels of high‐sensitivity C‐reactive protein (hs‐CRP), tumor necrosis factor (TNF), and interleukin 6 (IL‐6) may increase local inflammation in periodontal tissues, thereby accelerating disease progression [19, 20].

Therefore, prevention strategies, as suggested by the WHO [1], as well as integrated treatment plans targeting both obesity and periodontal health could have far‐reaching benefits for individuals and society as a whole [21]. Such an approach can only rely on a reliable definition of the link between obesity and periodontitis. Although several systematic reviews have explored the potential association between obesity and periodontitis, the question of whether obesity represents a consistent and independent risk factor for periodontitis remains unresolved. Previous reviews have yielded heterogeneous results due to differences in inclusion criteria, clinical definitions of obesity and periodontitis, and lack of subgroup analyses by age, region, or study design. For example, although Kim et al. [3] found an increased odds of periodontitis in subjects with obesity, especially among young adults, the overall heterogeneity remained high, and only observational studies were included. Similarly, Abu‐Shawish et al. [22] conducted a systematic review without meta‐analysis, and Martinez‐Herrera et al. [23] provided mainly a narrative synthesis. Furthermore, some reviews such as Khan et al. [24] focused exclusively on younger populations.

This systematic review and meta‐analysis aimed to critically appraise and quantitatively synthesize current evidence on the association between obesity and periodontitis in adults.

## 2 Methods

The protocol for this systematic review and meta‐analysis was developed in accordance with the PRISMA‐P (Preferred Reporting Items for Systematic Reviews and Meta‐Analyses Protocols) 2015 guidelines and was submitted and registered in the PROSPERO database (CRD42024581512).

### 2.1 Search Strategy and Database Screening

An electronic search of the English language literature was performed up to August 2024 on the following databases: PUBMED, SCOPUS, and Web of Science.

In addition, a direct search was also performed in the bibliographies of all reviewed articles.

The research process was carried out via a combination of mesh terms and free text words, combined using some Boolean operators (AND, OR). The following protocol was used for PUBMED: (“periodontitis” [MeSH] OR “periodontal diseases” [MeSH] OR “gingival disease”) AND (“oral cavity” OR “mouth”) AND (“obesity” [MeSH] OR “body mass index” [MeSH] OR “BMI” OR “overweight”). Search strategies for each specific database are available in Table S1.

### 2.2 Eligibility Criteria

#### 2.2.1 Inclusion Criteria

No publication year restriction was applied to the mentioned search. Studies were screened based on the following inclusion criteria: (1) English language; (2) cohort, case–control, observational retrospective, or longitudinal studies; (3) studies that have statistically evaluated the risk of people with obesity to develop periodontitis; (4) studies in adults (> 17 years old); (5) and to be eligible for inclusion in the meta‐analysis, the outcome had to be reported as odds ratio between obesity and periodontitis.

#### 2.2.2 Exclusion Criteria

The exclusion criteria were as follows: (1) systematic review and meta‐analysis; (2) studies not including information about the association between periodontitis and obesity; (3) studies not in English; and (4) studies in children (< 17 years old).

### 2.3 Focused PICO Question and Effect Measure

(P) Participants: patients with obesity (BMI > 30 kg/m^2^).

(I) Interventions: assessment of periodontitis in patients with obesity.

(C) Comparison between interventions: incidence of periodontal disease in patients with obesity versus patients without obesity.

(O) Outcome measures: correlation between obesity and periodontitis.

### 2.4 Studies Screening and Inclusion

Two authors (FE and LLR) independently screened retrieved citations by reading titles and abstracts. After considering inclusion and exclusion criteria, the authors came up with a list to screen for full‐text eligibility evaluation. The third author (GS) was involved in making a final decision in case of disagreements.

### 2.5 Data Extraction

Similarly, data extraction was undertaken by two reviewers (FE and LLR), independently, and results were compared and merged by using an ad hoc extraction sheet. In case of a discrepancy, articles were rescreened in a joint meeting with the third reviewer (GS). The extraction sheet in Excel format included the following fields: author, country, study design, sample size (*N*), age range, mean age, periodontal evaluation, obesity evaluation, and significant association (Y/N) (Table 1).

**TABLE 1:** Characteristics of included studies.

Author	Country	Study design	*N*	Age range	Mean age	Periodontal evaluation	Obesity evaluation	Significant association (Y/N)
Al‐Zahrani_2003	United States	BAS	13,665	> 18	NR	CAL > 3 mm PPD > 4 mm	BMI ≥ 30 kg/m^2^	Y
Alsalihi_2021	Baharein	BAS	354	> 18	42.9	CPI	WC BMI > 30 kg/m^2^	Y
Benguigui_2012	France	BAS	186	35–64	54	CAL > 4 mm PPD > 4 mm	BMI > 30 kg m^2^; WC > 102 cm in men WC > 88 cm in women.	Y
Dalla Vecchia_2005	Brazil	BAS	706	30–65	NR	CAL ≥ 5 mm	BMI ≥ 30 kg/m^2^	Y
de Castilhos_2012	Brazil	BAS	720	30	NR	CAL	BMI ≥ 30 kg/m^2^	Y
Ekuni_2008	Japan	BAS	618	18–24	NR	CPI > 2	BMI ≥ 30 kg/m^2^	Y
Ekuni_2014	Japan	Prospective cohort study	224	18–25	18.2	CPI > 2	BMI ≥ 30 kg/m^2^	Y
Haffajee_2009	United States	BAS	574	18–86	46.8	CAL e PPD	BMI ≥ 30 kg/m^2^	Y
Han_2010	Korea	BAS	1046	16–84	40.8	CPI	BMI‐WC‐VFA	Y
Jia_2023	China	BAS	112	NR	42.50 ± 7.42	CAL	BMI ≥ 30 kg/m^2^ e WHR	Y
Katagiri_2010	Japan	BAS	197	17–40	30	CPI	BMI ≥ 30 kg/m^2^	Y
Khader_2009	Jordan	BAS	340	18–70	NR	CAL > 3 mm PPD > 4 mm	BMI ≥ 30 g/m^2^	Y
Kim_2016	Korea	BAS	11,466	19–65	NR	CPI	BMI ≥ 30 g/m^2^	Y
Kongstad_2009	Denmark	BAS	1597	20–95	NR	CAL	BMI ≥ 30 kg/m^2^	Y
Kumar_2009	India	BAS	513	18–54	NR	CPI	BMI ≥ 30 kg/m^2^	Y
Liu_2023	China	Cross sectional study	6662	30–44	51.8	CAL	BMI ≥ 30 kg/m^2^ WC	Y
Saito_2001	Japan	BAS	643	19–79	NR	CAL e PPD	BMI ≥ 30 kg/m^2^	Y
Santos_2019	Brazil	BAS	236	18 ≥ 35	NR	CAL e PPD	BMI ≥ 30 kg/m^2^	Y
Saxlin_2010	Finland	BAS	1248	30–59	NR	CAL e PPD	BMI ≥ 30 kg/m^2^	Weak association

Abbreviations: BAS, before–after study; BMI, Body Mass Index; CAL, clinical attachment loss; CPI, Community Periodontal Index; PPD, probing pocket depth; WC, waist circumference; WHR, waist–hip ratio.

### 2.6 Assessment of Risk of Bias

The quality assessment and the risk of bias of the included studies were performed following the criteria of the Newcastle–Ottawa scale [25]. Two authors (FE and LLR) independently performed such assessment, and discrepancies were resolved in a joint meeting with the third reviewer (GS).

### 2.7 Statistical Analysis

Meta‐analyses were performed to calculate the pooled odds ratio and corresponding 95% confidence intervals (CIs), stratified by obesity and periodontitis status. The *I* ^2^ statistic classification was used to assess the heterogeneity of effect measures. Heterogeneity was categorized as low, moderate, or high for *I* ^2^ values of 25%, 50%, or 75%, respectively. An *I* ^2^ value greater than 50% indicated substantial heterogeneity. If heterogeneity exceeded 50%, the random effects method was applied; otherwise, the fixed effects method was used.

## 3 Results

### 3.1 Study Selection

**FIGURE 1:**
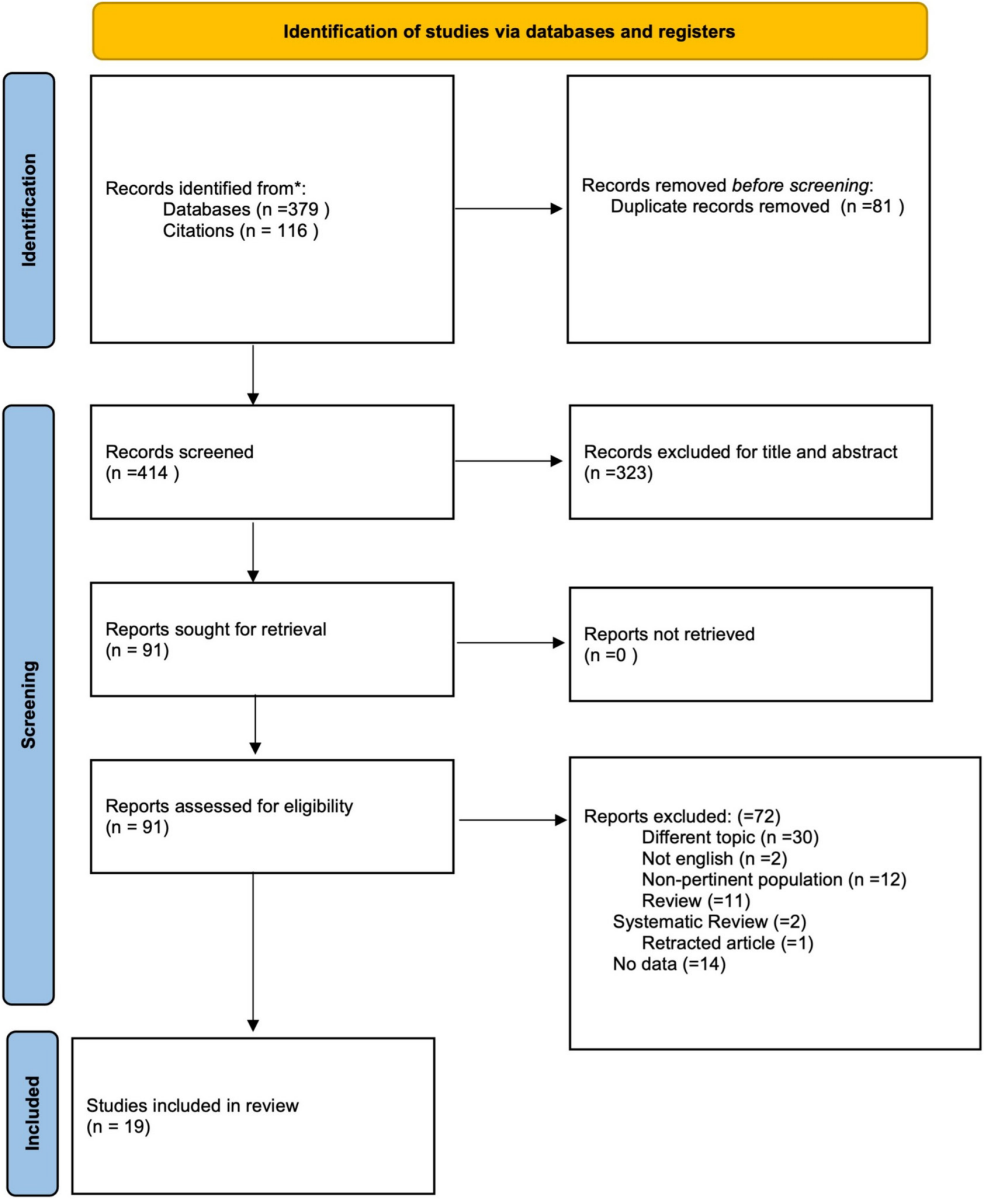
Flowchart of studies' selection process.

The electronic search retrieved a total of 379 articles from the electronic database search and 116 articles from the hand search published up to August 2024. After the removal of duplicates (81), title and abstract analysis was performed on 414 articles; 323 papers were excluded following the screening of titles and abstracts. Full text analysis was performed on the remaining 91 articles; 72 articles were further excluded (Table S2). Thus, 19 articles (Figure 1) were included in the final review and meta‐analysis [4, 8, 9, 10, 26, 27, 28, 29, 30, 31, 32, 33, 34, 35, 36, 37, 38, 39, 40].

### 3.2 Study Characteristics

The eligible studies (Table 1) were conducted from 2001 to 2023, in various countries around the world, including Baharein [27], Brazil [8, 29, 30], China [9, 10, 41], Denmark [37], Finland [40], France [28], India [38], Japan [31, 32, 35, 39], Jordan [4], Korea [34, 36], and the United States [26, 33].

The study population consisted of 41,110 patients, with a mean age of 42 years.

For the assessment of periodontal status, the Clinical Attachment Loss (CAL) index was used in five studies [9, 10, 29, 30, 37]. In seven studies [4, 8, 26, 28, 33, 39, 40], both indices, CAL and Probing Pocket Depths (PPD), were used. The Community Periodontal Index (CPI) index was used in seven studies [27, 31, 32, 34, 35, 36, 38].

In 15 studies [4, 8, 10, 26, 29, 30, 31, 32, 33, 35, 36, 37, 38, 39, 40], obesity was assessed according to the World Health Organization, with a body mass index (BMI) ≥ 30 kg/m^2^.

In three the studies [28, 34], both BMI and waist circumference (WC) were assessed; in the study of Jia et al. [9], obesity was assessed with BMI ≥ 30 kg/m^2^ and waist–hip ratio (WHR).

### 3.3 Risk of Bias

**FIGURE 2:**
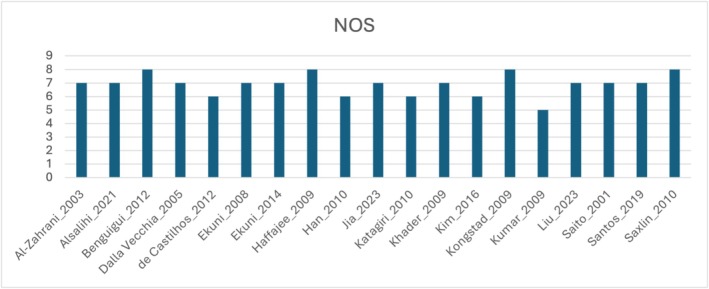
Study quality as assessed by the Newcastle Ottawa Scale (NOS).

Of the 19 studies, 14 [4, 8, 9, 10, 26, 27, 28, 29, 31, 32, 33, 37, 39, 40] were evaluated as *very good* and 5 [30, 34, 35, 36, 38] were evaluated as *good* (Figure 2).

### 3.4 Meta‐Analysis

Heterogeneity results showed high heterogeneity among studies (*I* ^2^ = 87.90%) Heterogeneity among studies was further investigated by the leave‐one‐out method (Figure S1), which did not show differences in SMD estimation.

**FIGURE 3:**
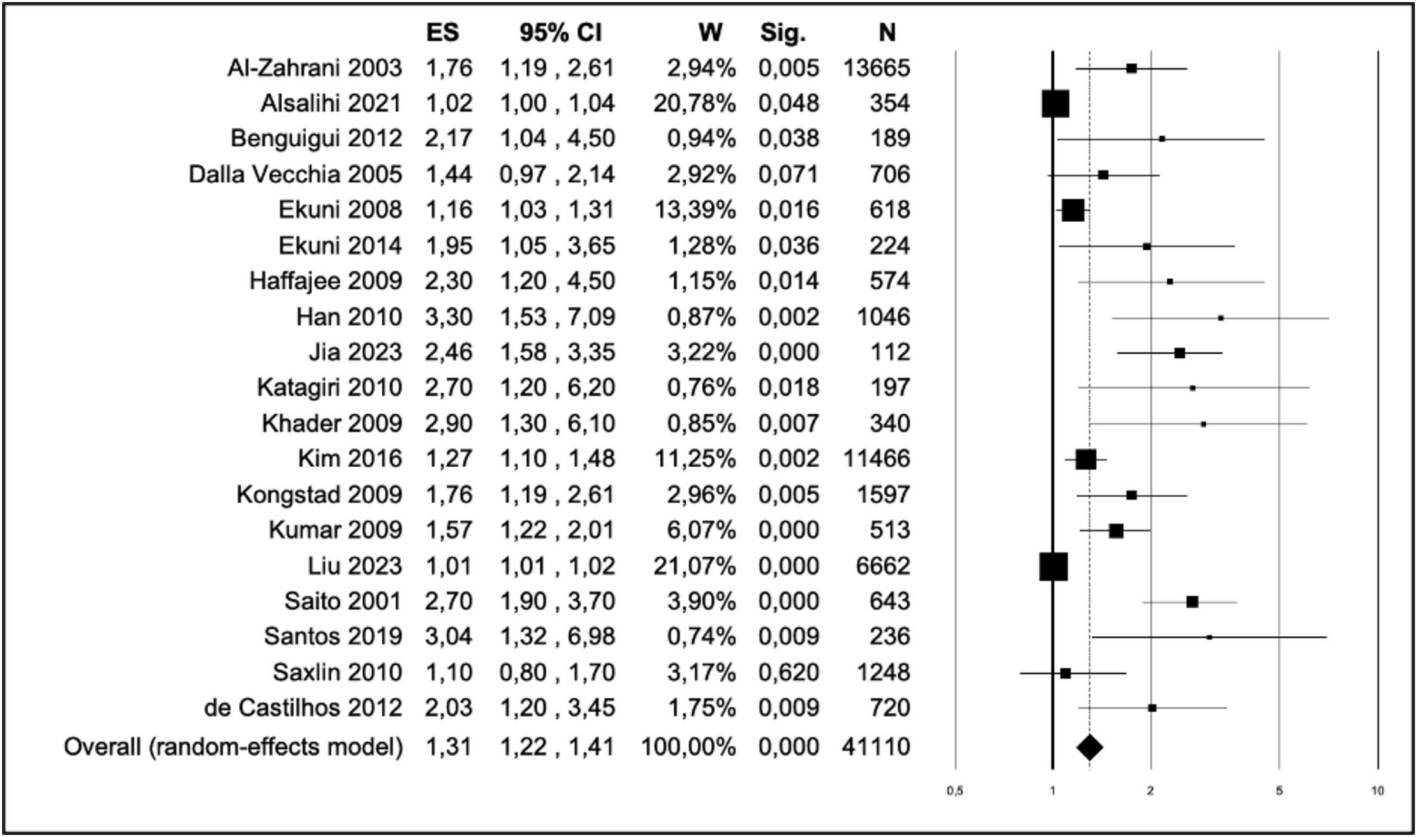
Forest plot of the risk of periodontitis for obesity.

The overall was 1.31, 95% CI of 1.22–1.41, and a *p* value of 0.00 (Figure 3).

## 4 Discussion

The objective of this systematic review and meta‐analysis was to evaluate if obesity is a risk factor for periodontitis. The synthesis of the evidence from 19 studies with a total of 41,110 participants demonstrated a strong and consistent connection: Individuals classified with obesity (BMI ≥ 30 kg/m^2^) had an odds ratio (OR) of 1.31 (95% CI: 1.22–1.41, *p* < 0.001), which means they were 31% more likely to have periodontitis. This connection also makes sense biologically. Obesity‐related inflammation promotes periodontitis because fat cells release numerous inflammatory factors such as TNF‐α, IL‐6, and CRP, which create chronic inflammation in the body and may intensify local inflammatory cascades within periodontal tissues, thereby fostering tissue damage and bone loss. Also proposed is increased oxidative stress in gingival tissues in the context of greater obesity as contributing to periodontal tissue breakdown [30, 32, 35, 37].

The effect of obesity may also alter the production and release of key immune cells, such as neutrophils—the primary innate immune responders in periodontal tissues—as well as T and B lymphocytes, which are responsible for cellular and humoral immunity [8].

Obesity may affect the production and release of certain crucial immune cells like neutrophils, which are the acute innate immune responders within the periodontal tissues, as well as T and B lymphocytes, which mediate cellular and humoral immunity. These alterations could hamper the control of bacterial biofilms, resulting in an exacerbated inflammatory response within periodontal tissues. One interesting aspect that stands out from the literature reviewed is that of gender and hormonal factors. Some authors have noted that young women with obesity seem to present an increased 80% greater risk than men of developing periodontal lesions [10, 27, 29, 35]. This heightened risk is thought to stem from hormonal changes, particularly the estrogen and progesterone rise during certain phases of the menstrual cycle, which has been shown to increase the expression of inflammatory cytokines in gingival tissues, leading to worsened periodontal inflammation [27]. Also notable is the age in this population group affected. Several studies noted a relatively young mean or median age for obesity with periodontitis diagnosis around 46.8 years [10, 26, 31, 33, 34, 35, 38]. This raises a concern regarding the hypothesis supporting obesity as an accelerating factor in causing periodontitis. Our analysis showed moderate to high heterogeneity across studies despite these findings. This could arise from differences pertaining to study methodology, definition of obesity and periodontitis, and adjustments for confounders. Subgroup and sensitivity analyses did confirm a robust association across age groups, definitions of obesity (BMI vs. waist circumference), and periodontal measures such as CAL/PD/BOP. Most studies were assessed to have a moderate risk of bias, with predominately confounding and diagnostic inconsistency being major causes. The overall certainty of evidence using GRADE was moderate, which supports the findings but also indicates a need for more prospective and interventional research for better quality evidence focused on causality. To sum up, this study strongly underlined the association between periodontitis and obesity, drawing attention from dental professionals to general health practitioners.

Tailored preventive measures are required that address central obesity alongside other components of metabolic syndrome. Targeting controllable trends in periodontal disease might help lower periods of decline due to retreating changes witnessing reversal trends outside metropolitan areas where prevalence made clinicians rethink their refresher training programs structured around treatment and recovery viewed far too optimistic computation.

## 5 Conclusion

The current systematic review and meta‐analysis show that obesity correlates with having an increased risk of periodontitis (OR = 1.31; 95% CI: 1.22–1.41). Although there is supporting evidence for a strong association, more longitudinal and interventional studies are needed to explain the causal mechanisms involved. This could impact research aimed at determining if combined preventive approaches for obesity would also benefit periodontal health.

## Author Contributions

**Fariba Esperouz:** study design, data collection, manuscript writing. **Lucio Lo Russo:** study design, data collection and analysis. **Gaetano Serviddio:** manuscript revision. **Domenico Ciavarella:** manuscript writing. **Mauro Lorusso:** data collection and analysis. **Claudio Di Gioia:** manuscript writing.

## Conflicts of Interest

The authors declare no conflicts of interest.

## Supporting information


**Table S1:** Search strategies.
**Table S2:** Reason to exclusion.
**Figure S1:** Leave one out method.
