# Supplementary material for: Is Obesity a Risk Factor for Periodontitis? A Systematic Review and Meta‐Analysis
Source: Obes Rev. 2025 Sep 16;27(2):e70020. doi: 10.1111/obr.70020 (PMC12812498; doi:10.1111/obr.70020)

Title: Is Obesity a risk factor for periodontitis: Systematic review and Meta-analysis

Authors: Esperouz Fariba[a], Ciavarella Domenico[a], Di Gioia Claudio, Serviddio Gaetano[b],Lorusso Mauro[a], Lo Russo Lucio[a]

Affiliations: a. Department of Clinical and Experimental Medicine, School of Dentistry, University of Foggia, 71121 Foggia

b. Department of Medical and Surgical Sciences, University of Foggia, Foggia, Italy.

Keywords: Obesity, Periodontitis, Body Mass Index, BMI, Probing depth, Prevention, Oral Health

Acknowledgements : This research was funded by the Health Extended Alliance for Innovative Therapies, Advanced Lab-research, and Integrated Approaches of Precision Medicine—acronimo HEAL ITALIA (grant number PE_00000019—CUP D73C22001230006)" – PNRR MUR – M4C2 – Investimento 1.3 - Public Call "Partenariati Estesi" - D.D. n. 341/2022." - SPOKE 1;

Corresponding author: Dr. Esperouz Fariba

Via Rovelli 48, 71122 Foggia, fariba.esperouz@unifg.it, Tel: 0881588082

Declaration of Competing Interest

The authors declare that they have no known competing financial interests or personal relationships that could have appeared to influence the work reported in this paper.

Running title : Obesity and Periodontitis: A Systematic Review and Meta-analysis

Table S1 Search strategies

Database	Search strategy
PubMed	(("periodontitis"[MeSH Terms] OR "periodontal diseases"[MeSH Terms] OR "gingival disease") AND ("oral cavity" OR "mouth") AND ("obesity"[MeSH Terms] OR "body mass index"[MeSH Terms] OR "BMI" OR "overweight"))
Scopus	(("periodontitis" [MeSH] OR "periodontal diseases" [MeSH] OR "gingival disease") AND ("oral cavity" OR "mouth") AND ("obesity" [MeSH] OR "body mass index" [MeSH] OR "BMI" OR "overweight"))
Web of science	(("periodontitis"[MeSH] OR "periodontal diseases"[MeSH] OR “gingival disease”) AND (“oral cavity” OR "mouth") AND ("obesity"[MeSH] OR "body mass index"[MeSH] OR “BMI” OR “overweight”))

Table S2 : Reason to exclusion

ID	MOTIVATION
Abdo_2013	Different topic
Adam_2023	Systematic review
Adegun_2011	No data for meta-anlysis
Alhabashneh_2015	Different topic
Andriankaja_2010	Different topic
Bandiwadekar_2020	Different topic
Bastos_2018	Different topic
Bharti_2009	Review
Borges_2007	Not english
Boukeng_2023	Non-pertinent population: pediatric
Budunelli_2014	Non-pertinent population:no men
Campos_2020	Different topic
Charupinijkul_2021	No data for meta-anlysis
Chatzopoulos_2016	Different topic
Chen_2021	No data for meta-anlysis
Cortelli_2021	Review
Cullinan_2009	Review
D'Aiuto_2008	Different topic
de Andrade_2020	Different topic
Dumitrescu_2008	Retracted article
Dumitrescu_2009	No data for meta-anlysis
Franchini_2011	Non-pertinent population: pediatric
Fukui_2012	Different topic
Gambino_2020	No data for meta-anlysis
Gomes-Filho_2016	Different topic
Goodson_2019	Non-pertinent population: pediatric
Gurav_2014	Systematic review
Hedge_2019	Review

Husham Ali	Non-pertinent population:no men
Ikbariyeh_2022	Different topic
Iwasaki_2015	Different topic
Jepsen_2020	Review
Kaye_2016	Non-pertinent population:no women
Khemiss_2021	No data for meta-anlysis
Kim_2017	Different topic
Kim_2019	Different topic
Kotin_2021	Different topic
Kumar_2016	Different topic
Kushiyama_2009	Different topic
Lamster_2017	Review
Levine_2013	Review
Li_2009	Different topic
Li_2022	Different topic
Linden_2007	Non-pertinent population:no women
Maisel_2012	Different topic
Marcenes_2003	Different topic
Marro_2021	Non-pertinent population: pediatric
Matsushita_2015	Review
Mikami_2021	Different topic
Morita_2009	Different topic
Musskopf_2016	Different topic
Nascimento_2017	No data for meta-anlysis
Nesbitt_2010	Different topic
Nilsen_2023	Different topic
Palle_2013	No data for meta-anlysis
Pataro_2011	Non-pertinent population:no men
Peruchi_2015	No data for meta-anlysis
Pham_2018	Different topic
Prpic_2010	No data for meta-anlysis
Rahman_2023	No data for meta-anlysis

Roa_2018	Review
Saito_2005	Non-pertinent population:no men
Saito_2023	Different topic
Salamonowicz_2019	Review
Sarlati_2008	No data for meta-anlysis
Sheiham_2002	Different topic
Sonoda_2018	Non-pertinent population:no women
Suvan_2013	No data for meta-anlysis
Suvan_2020	No data for meta-anlysis
Vallim_2020	No data for meta-anlysis
Wang_2021	Not english
Yang_2023	Different topic
Ylöstalo_2007	No data for meta-anlysis
Zhao_2022	Review

Fig. S1 Leave one out method

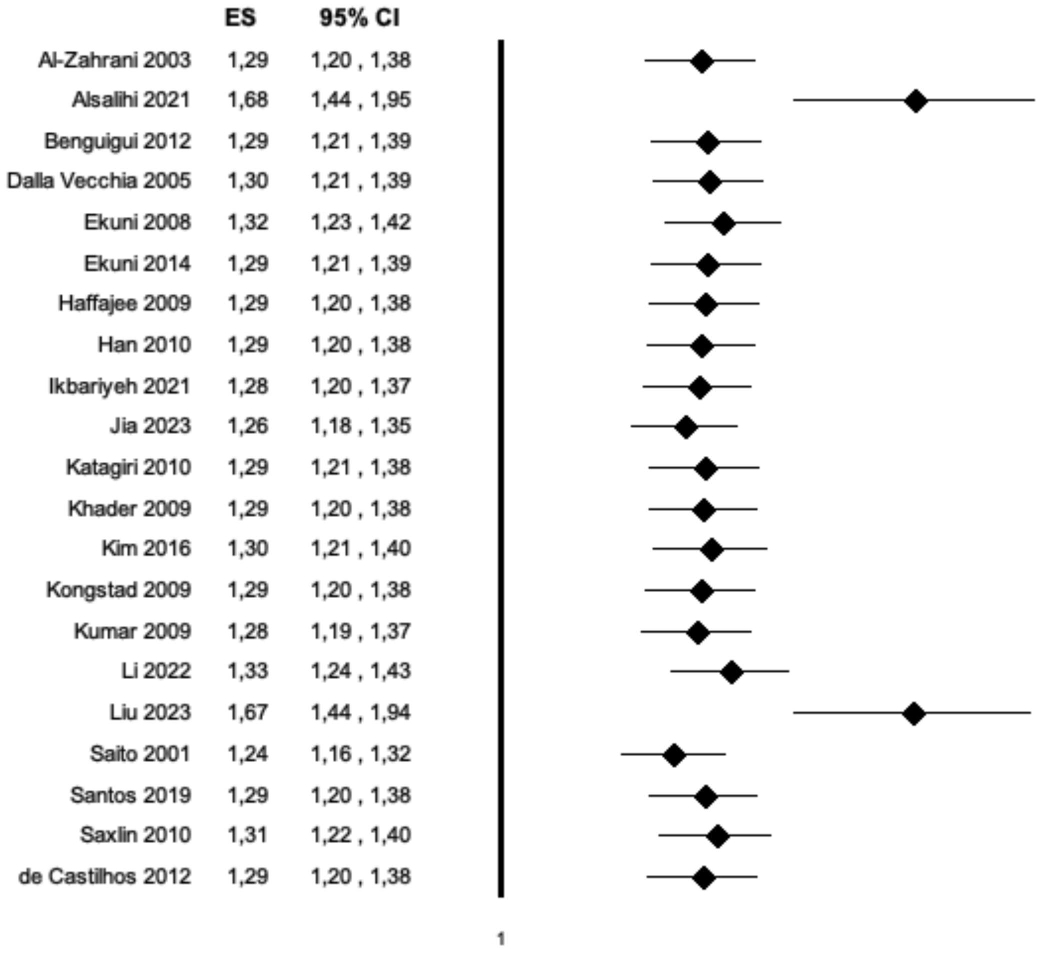

Supplement: Supplementary file 1 — Table S1: Search strategies. Table S2: Reason to exclusion. Figure S1: Leave one out method. [file OBR-27-e70020-s001.pdf]
